# Effect of Cd Doping on the Gas-Sensitive Properties of ZnSn(OH)_6_

**DOI:** 10.3390/ma18133176

**Published:** 2025-07-04

**Authors:** Yufeng Wen, Yanlin Yu, Huaizhang Gu, Guilian Wang, Fangqiang Yuan

**Affiliations:** 1 School of Science, Kaili University, Kaili 556011, China; kluwenyf@163.com (Y.W.); yuyanlin_121@163.com (Y.Y.); guhzh666@163.com (H.G.); 2 School of Mathematical Sciences and Physics, Jinggangshan University, Ji’an 343009, China; 3 School of Electronic and Electrical Engineering, Shanghai University of Engineering Science, Shanghai 201620, China; wglwrc2016@126.com; 4 School of Electronic and Electrical Engineering, Zibo Vocational University, Zibo 255300, China

**Keywords:** ZnSn(OH)_6_, hydrothermal method, gas-sensitive properties, Cd doping, density functional theory

## Abstract

The influence of Cd doping on the performance of ZnSn(OH)_6_ (ZHS) as a gas sensor was systematically investigated through experimental and theoretical approaches. ZHS and Cd-doped ZHS samples were synthesized using the hydrothermal method. The microstructures of pure and Cd-doped ZHS were characterized using various techniques. The results revealed that the pure ZHS sample exhibits good crystallinity and an octahedral morphology with particle sizes ranging from 800 to 1900 nm. After Cd doping, the particle size range was decreased to 700–1500 nm. A systematic investigation of the gas-sensing properties revealed that Cd-doped ZHS exhibits superior sensing performance toward ethanol gas compared to pure ZHS. Under operating conditions of 240 °C, 100 ppm concentration, and 30% relative humidity, the response of ZHS to ethanol gas exhibited a significantly higher value compared to other tested gases. After Cd doping, the response approximately doubled. Density functional theory calculations of electronic structures revealed that the enhanced ethanol sensing mechanism of Cd-doped ZHS is attributed to the narrowed band gap caused by Cd doping, which increases electron concentration and enhances $O^{-}$ ion adsorption on the surface.

## 1. Introduction

ZnSn(OH)_6_ (ZHS) is a promising semiconductor material with broad applications in catalysis [1,2,3], batteries [4,5], flame retardants [6,7], and gas sensors [8,9]. However, ZHS’s performance is significantly limited due to its large bandgap (approximately 4 eV [10,11,12]) and high electrical resistivity, restricting its practical applications. The gas-sensitive properties of semiconductors can be improved via morphology control [1,2,3,4,5,6,7,8,9,13,14,15], introducing defects [16,17], doping [10,11,12,18,19], forming heterojunctions [20,21], and UV irradiation [22,23]. Among these methods, doping emerges as a significant and effective strategy for modulating resistivity and carrier concentration.

ZHS demonstrates gas-sensitizing properties for ethanol detection [24,25,26,27,28]. The high electrical resistivity of ZHS poses challenges for device fabrication. ZHS’s highly symmetric lattice structure facilitates the investigation of gas sensitivity mechanisms and the relationship between morphology and crystal growth orientation. ZHS doped with Al and Bi elements has been synthesized [27,28]. Studies have shown that Al substitutes Zn atoms in ZHS, while Bi exhibits similarities to Sn atoms in terms of atomic radius and electron configuration [27,28]. Due to the higher valence electron density of Al and Bi compared to Zn and Sn, respectively, their doping enhances the electron concentration. As an n-type semiconductor, ZHS exhibits decreased electrical resistance with increased electron concentration. Simultaneously, the gas-sensing properties of ZHS are improved due to the abundance of free electrons.

In the chemical periodic table, Al and Bi are the adjacent main-group elements located to the right of Zn and Sn, respectively. Their valence electrons are one more than those of their respective parent elements, leading to an increase in the electron concentration of ZHS. Cd, a member of the same group as Zn and located directly below it, shares a similar valence electron configuration. Given that Cd and Zn belong to the same group, could Cd doping, similar to Bi and Al doping, increase the electron concentration of the material and thereby improve the gas-sensing performance of ZHS? Motivated by this question, we will experimentally and theoretically investigate the effect of Cd doping on the gas sensitivity of ZHS in this paper. The investigation will employ the hydrothermal synthesis method and characterization techniques, including X-ray diffraction (XRD), scanning electron microscopy (SEM), transmission electron microscopy (TEM), energy-dispersive X-ray spectroscopy (EDX), and X-ray photoelectron spectroscopy (XPS), as well as density functional theory (DFT) calculations.

## 2. Experiments and Calculations

### 2.1. Synthesis of ZHS and Cd-Doped ZHS (Cd@ZHS)

**Figure 1:**
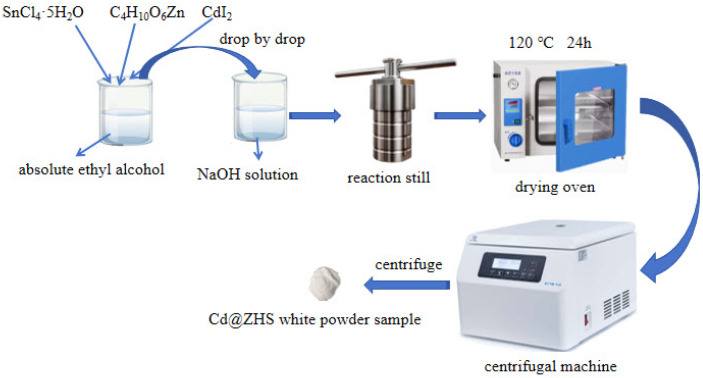
Synthesis process of the Cd@ZHS sample.

A total of 0.440 g of (CH_3_COO)_2_Zn·2H_2_O and 0.701 g of SnCl_4_·5H_2_O were dissolved in 30 mL of ethanol, then the mixture was stirred for 30 min to prepare solution A. A toal of 1.000 g of NaOH was dissolved in 30 mL of deionized water, then the mixture was stirred for 30 min to prepare solution B. Solution A was slowly added dropwise to solution B under magnetic stirring, and the mixture was stirred for an additional 30 min to prepare solution C. A total of 60 mL of the white solution C was transferred into a 100 mL polytetrafluoroethylene-lined autoclave and heated at 120 °C for 24 h. The precipitate was collected from the solution, washed, and centrifuged four times using deionized water and alcohol. Finally, the precipitate was dried in an oven at 60 °C for 24 h to prepare sample ZHS. The synthesis process for Cd@ZHS was identical to the preparation of ZHS. Sample Cd@ZHS was prepared by dissolving 0.440 g of (CH_3_COO)_2_Zn·2H_2_O, 0.701 g of SnCl_4_·5H_2_O, and 0.022 g of CdI_2_ in 30 mL of ethanol, as shown in Figure 1.

### 2.2. DFT Calculations

To investigate the electronic structures of ZHS and Cd-doped ZHS, DFT calculations were performed using the CASTEP software (cost-free academic version) package [29], employing projector augmented wave pseudopotentials [30,31]. The exchange-correlation functional was described using the Perdew–Burke–Ernzerhof (PBE) generalized gradient approximation (GGA) [32]. A plane wave cutoff energy of 571.4 eV was selected for the calculations. A 2×2×2 Monkhorst–Pack k-point mesh [33] was used for Brillouin zone integration. Geometry optimization was performed using the Broyden–Fletcher–Goldfarb–Shanno (BFGS) optimization algorithm [34], with convergence criteria including a total energy change of $10^{-6}$ eVatom, maximum atomic displacement of 0.001 Å, maximum force of 0.03 eV/Å, and maximum stress of 0.05 GPa. For Cd doping, a 2×2×2 supercell model was established, containing 32 Zn, 32 Sn, and 192 O atoms. A single Zn atom was substituted by a Cd atom, resulting in a Cd concentration of 0.39 at.%.

## 3. Results and Discussion

### 3.1. Characterization

**Figure 2:**
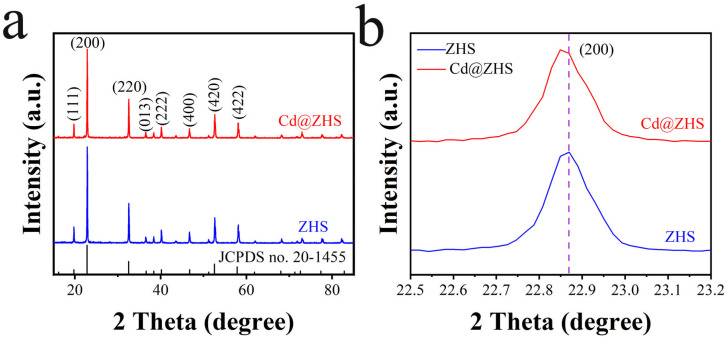
(**a**,**b**) XRD patterns of ZHS and Cd@ZHS samples.

The XRD patterns of the ZHS and Cd@ZHS samples in Figure 2 match the ZHS standard card (20-1455) without extraneous peaks, confirming their identification as ZHS. Cd impurities caused a leftward shift in the characteristic peaks, indicating an increase in lattice constants. Cd has a larger atomic radius than Zn but smaller than Sn. Cd substitution for Zn increases lattice constants, as evidenced by the peak shift to the left in the XRD patterns. Conversely, Cd substitution for Sn would cause a rightward peak shift, inconsistent with our experimental results. XRD characterization shows that the space group of ZHS is Pn-3 (no. 201), with lattice parameters a=b=c=7.9347 Å and α=β=γ=90°. This indicates that the crystallographic directions *a*, *b*, and *c* are equivalent, resulting in octahedral faces with crystallographic indices (200), ($\bar{2}$00), (020), (0$\bar{2}$0), (002) and (00$\bar{2}$). After Cd doping, the lattice constant was determined to be 7.9414 Å based on the XRD result of the Cd@ZHS sample.

**Figure 3:**
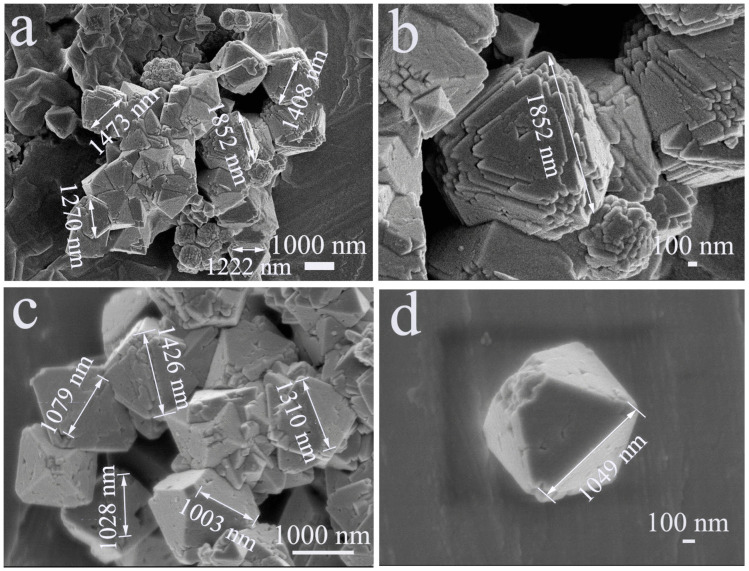
(**a**,**c**) SEM and (**b**,**d**) HRSEM of ZHS and Cd@ZHS. The subplot (**b**) is from Ref. [38].

**Figure 4:**
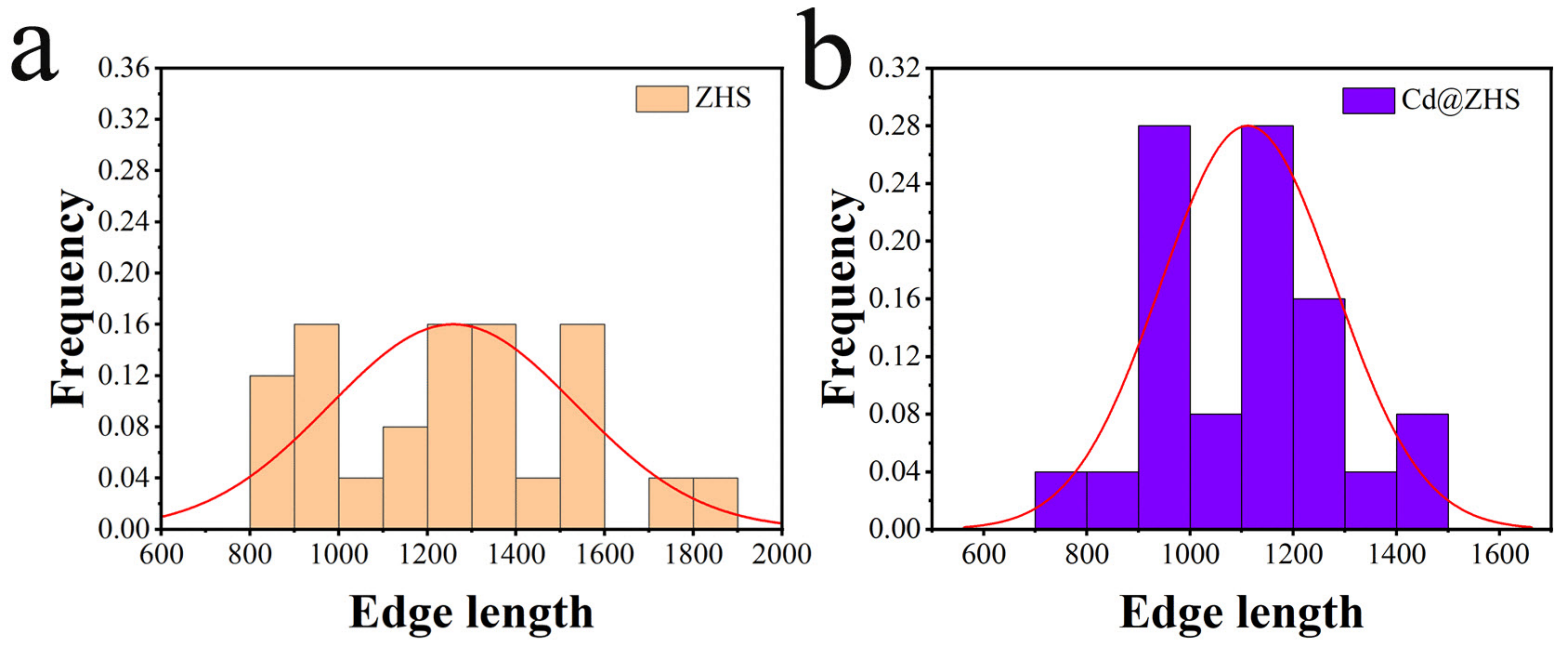
Particle size distribution for both (**a**) ZHS and (**b**) Cd@ZHS samples.

The SEM and HRSEM images in Figure 3 show that the synthesized ZHS and Cd@ZHS samples exhibit octahedral morphologies with step-like stripes or small holes on the surface. The edges and corners of the octahedra are absent due to their high surface free energy, which leads to dissolution and minimizes the system’s energy [35]. The particle size distributions for both ZHS and Cd@ZHS samples are shown in Figure 4. The ZHS sample has particle sizes ranging from 800 to 1900 nm, while Cd@ZHS sample exhibits sizes of 700–1500 nm, indicating that Cd doping reduces particle sizes. This reduction is attributed to the effects of doping on crystal growth and lattice distortion, which increase system energy and hinder crystal growth [36,37].

**Figure 5:**
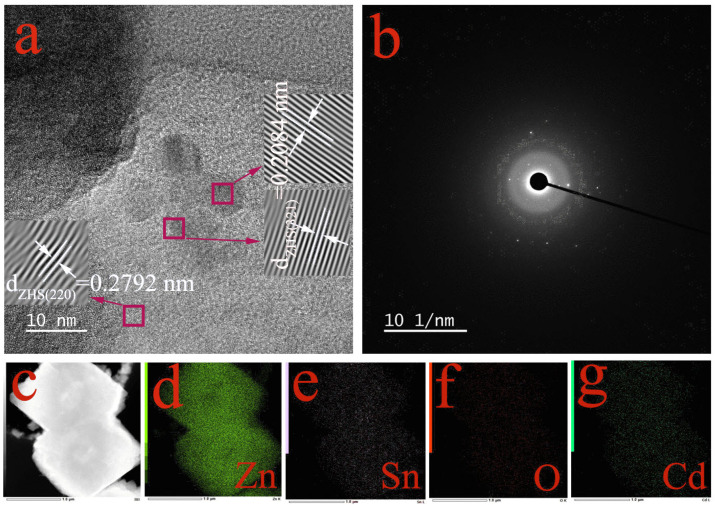
(**a**) HRTEM, (**b**) SEAD, (**c**) TEM, and (**d**–**g**) EDX mapping of Cd@ZHS.

The HRTEM image in Figure 5a reveals that the interplanar spacing of (220)/(321) for Cd@ZHS is 0.2792/0.2084 nm, which closely matches the value of 0.2760/0.2084 nm reported in the XRD standard card for ZHS. The selected area electron diffraction (SEAD) image in Figure 5b demonstrates that the Cd@ZHS sample is a single crystal. Figure 5d–g are EDX mapping images, which reveal that the elements Zn, Sn, O, and Cd in the sample are uniformly distributed, rather than being a simple mixture of the four elements or other compounds. The resulting sample is unambiguously confirmed to be ZHS with successful Cd doping.

**Figure 6:**
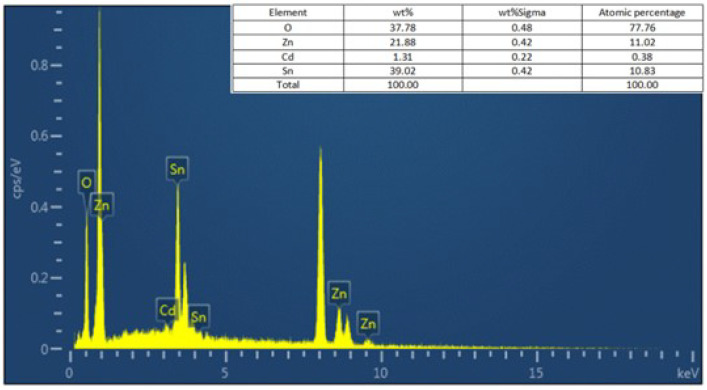
EDS analysis of Cd@ZHS sample.

Figure 6 shows the energy dispersive spectroscopy (EDS) of the Cd@ZHS sample. The presence of Zn, Sn, O, and Cd elements is observed, with atomic concentrations of 11.02 at.%, 10.83 at.%, 77.76 at.%, and 0.38 at.%, respectively. Based on the atomic proportions, the following can be determined: (I) The Zn-to-Sn ratio is approximately 1:1. (II) The oxygen content exceeds sevenfold that of Zn or Sn. This is attributed to the material’s exposure to ambient air, which leads to adsorbed oxygen on its surface. (III) Cd atoms account for approximately 0.38 at.% of the total atoms. This is owing to the adsorbed oxygen on the material’s surface, which reduces the proportions of other elements, and the low efficiency of Cd incorporation into the material during synthesis. The low Cd incorporation efficiency is likely due to lattice distortion caused by impurities, which increases lattice energy and prevents some Cd atoms from integrating into the lattice.

**Figure 7:**
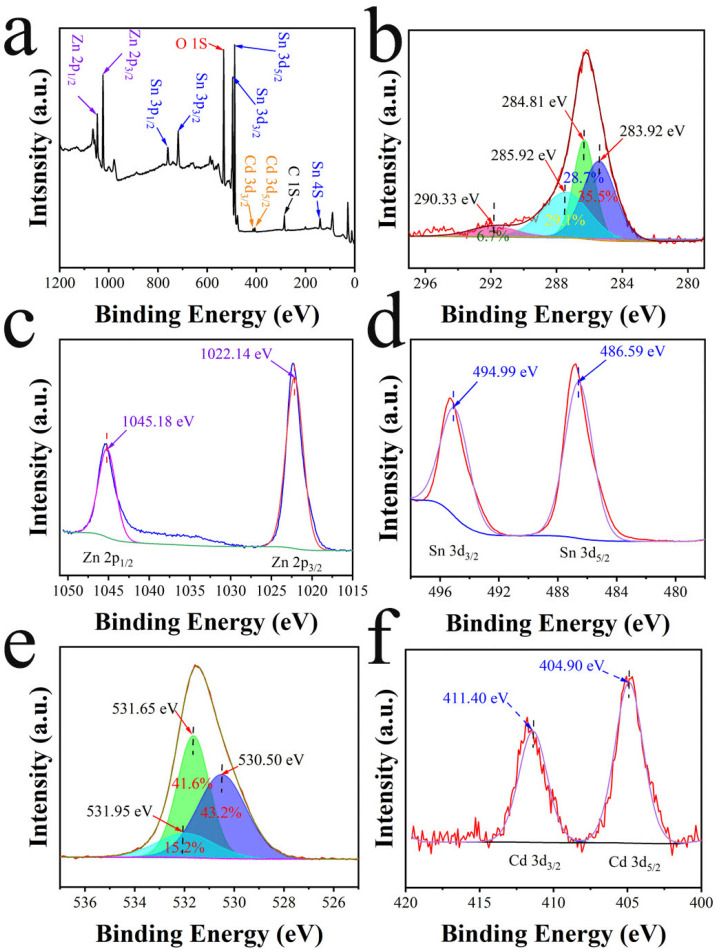
XPS spectra of Cd@ZHS: (**a**) survey spectra, (**b**) C 1s, (**c**) Zn 2p, (**d**) Sn 3d, (**e**) O 1s, and (**f**) Cd 3d.

The XPS survey spectrum in Figure 7a reveals the presence of Zn, Sn, O, Cd, and C in the Cd@ZHS sample. The high-resolution spectrum of C 1s in Figure 7b shows four peaks at 283.92, 284.81, 285.92, and 290.33 eV, corresponding to C–C, C–O, C=O, and O–C=O, respectively [39]. The Zn 2p spectrum in Figure 7c shows two characteristic peaks at 1045.18 eV (Zn 2$p_{1/2}$) and 1022.14 eV (Zn 2$p_{3/2}$). The Sn 3d spectrum in Figure 7d exhibits two characteristic peaks at 494.99 eV (Sn 3$d_{3/2}$) and 486.59 eV (Sn 3$d_{5/2}$). The Cd 3d spectrum in Figure 7f shows two characteristic peaks at 411.40 eV (Cd 3$d_{3/2}$) and 404.90 eV (Cd 3$d_{5/2}$). The O 1s spectrum in Figure 7e shows three characteristic peaks: vacancy oxygen ($O_{v}$, 530.50 eV, 43.2%), lattice oxygen ($O_{l}$, 531.65 eV, 41.6%), and adsorbed oxygen ($O_{a}$, 531.95 eV, 15.2%). Surface adsorbed oxygen significantly influences resistance changes and enhances gas-sensing properties [40,41].

**Figure 8:**
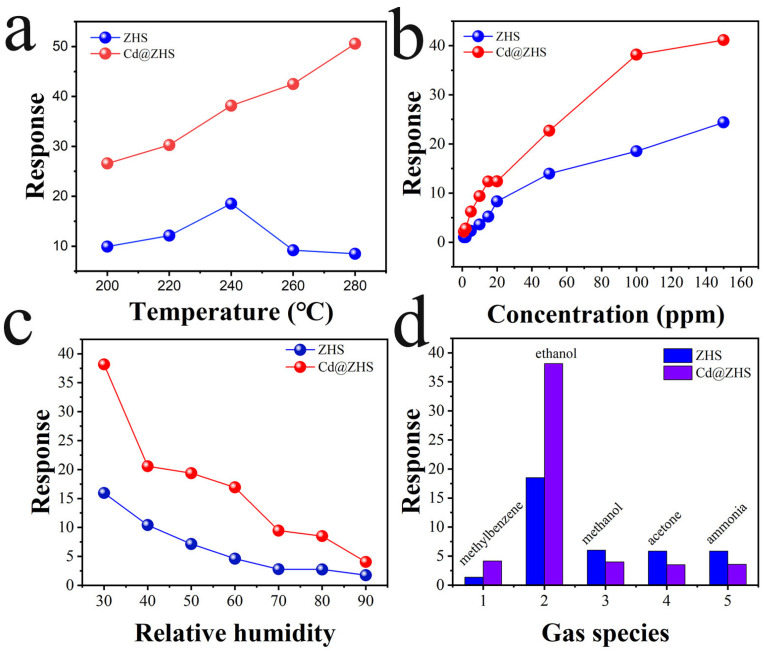
(**a**) Temperature-dependent response values of ZHS and Cd@ZHS to 100 ppm ethanol under 30% relative humidity; (**b**) response characteristics of ZHS and Cd@ZHS to varying ethanol concentrations under 30% relative humidity and 240 °C operating conditions; (**c**) humidity-dependent sensing behavior of ZHS and Cd@ZHS for 100 ppm ethanol at the optimal operating temperature of 240 °C; (**d**) Selectivity evaluation of ZHS and Cd@ZHS toward 100 ppm target gases under 240 °C and 30% relative humidity conditions.

The response is defined as $R_{a}/R_{g}$, where $R_{a}$ denotes the sensor resistance exposed in air, and $R_{g}$ represents the resistance under exposure to a specific concentration of the test gas. Considering that the decomposition temperature of ZHS is approximately 300 °C [42], the maximum test temperature was set at 280 °C in this study. As shown in Figure 8a, the response characteristics of ZHS and Cd@ZHS sensors to 100 ppm ethanol under 30% relative humidity reveal that the ZHS sensor’s response increases and then decreases with rising test temperature, reaching a maximum at 240 °C. In contrast, the Cd@ZHS sensor’s response consistently increases with temperature, surpassing that of the ZHS sensor at all tested temperatures. Figure 8b shows the response of ZHS and Cd@ZHS sensors to ethanol concentrations under 30% relative humidity and 240 °C. Both sensors exhibit increasing responses with ethanol concentration, with Cd@ZHS consistently outperforming ZHS. The response increases significantly in the 0–20 ppm range and then slows from 20 to 150 ppm. The response trend of ZHS and Cd@ZHS sensors to 100 ppm ethanol under optimal operating temperature, as shown in Figure 8c, indicates that both sensors’ responses decrease with increasing relative humidity. Additionally, the Cd@ZHS sensor consistently demonstrates a higher response than the ZHS sensor across all temperatures. The responses of ZHS and Cd@ZHS sensors to 100 ppm different gases under 240 °C and 30% relative humidity conditions, as shown in Figure 8d, demonstrate that both sensors exhibit significantly higher responses to ethanol compared to methylbenzene, methanol, acetone, and ammonia. Notably, the Cd@ZHS sensor’s response to ethanol is approximately twice that of the ZHS sensor, indicating that Cd doping significantly enhances sensing performance and ethanol selectivity.

**Figure 9:**
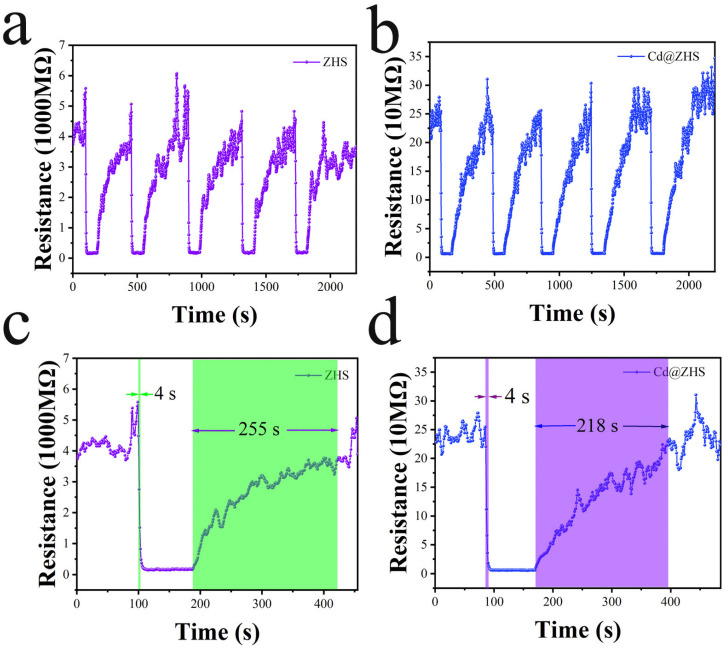
(**a**,**b**) Repeatability test of ZHS and Cd@ZHS sensors; (**c**,**d**) Adsorption and desorption process of ZHS and Cd@ZHS sensors for 100 ppm ethanol.

The repeatability tests of ZHS and Cd@ZHS sensors, as shown in Figure 9a,b, demonstrate strong reproducibility for ethanol detection. After five adsorption–desorption cycles, the ethanol response of the ZHS sensor exhibited a slight attenuation, while no attenuation was observed for the Cd@ZHS sensor. Cd doping significantly reduces the resistance of the ZHS sensor, which reduces its power consumption. As shown in Figure 9c,d, the adsorption and desorption processes of ZHS and Cd@ZHS sensors for 100 ppm ethanol reveal that both sensors exhibit a short adsorption time of 4 s. Additionally, Cd doping reduces the desorption time of the ZHS sensor by 37 s. Consequently, the Cd@ZHS sensor exhibits rapid response to ethanol gas, enabling more convenient and efficient real-time detection of the specific gas.

**Figure 10:**
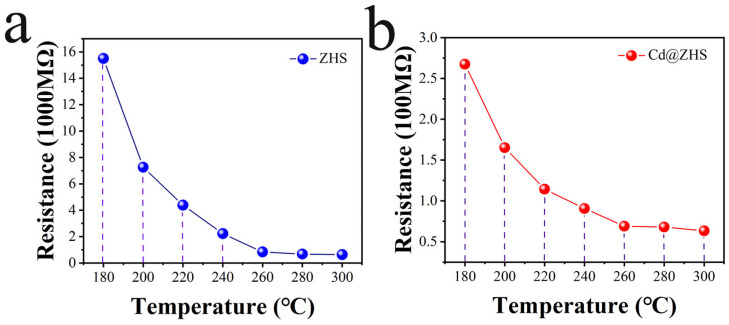
Resistance of (**a**) ZHS and (**b**) Cd@ZHS under different temperatures.

The resistance of ZHS and Cd@ZHS sensors, as shown in Figure 10, decreases with increasing temperature. Additionally, the rate of resistance change with temperature gradually decreases and approaches zero. These phenomena occur because, as temperature increases, electrons are transferred from the highest occupied orbitals to the lowest unoccupied orbitals, increasing the concentration of nearly free electrons, thereby reducing resistance. However, this constant electron transition decreases the energy of the highest occupied orbitals in the valence band and increases the energy of the lowest unoccupied orbitals in the conduction band, resulting in a greater difference (ΔE). Since the probability of electron transitions is proportional to $e^{-\Delta E/\hbar }$, this probability decreases with increasing temperature, leading to a reduction in the rate of resistance change with temperature. When almost all electrons in one energy band are transferred to higher energy levels, further temperature increases no longer reduce resistance within a certain temperature range. In fact, temperature increases may even cause a slight resistance increase. These findings occur because electrons no longer undergo transitions, resulting in a constant concentration of nearly free electrons. However, as temperature rises, lattice vibrations intensify, increasing the electron scattering and causing a slight resistance increase. Comparing the resistance in Figure 10a,b reveals that Cd doping significantly reduces the resistance of ZHS by more than one order of magnitude. The outermost electron is held less tightly as the atomic radius increases, making it easier to lose. Due to its larger radius, Cd has a weaker binding effect on its outermost electrons compared to Zn. Cd doping enhances the free electron concentration, thereby reducing the electrical resistivity of ZHS. Additionally, Cd substitution for Zn increases the lattice constants and causes a left shift in the XRD characteristic peaks. Conversely, since Cd has only half the outermost electrons of Sn, Cd substitution for Sn reduces the free electron concentration, increasing its electrical resistivity. Therefore, it can be concluded that Cd impurity atoms preferentially replace Zn atoms in ZHS.

**Figure 11:**
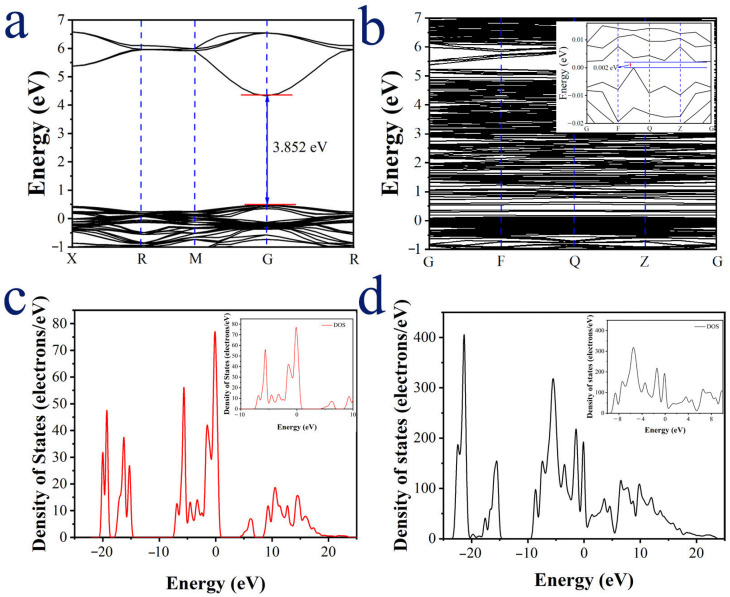
(**a**) Band structure and (**c**) density of states (DOS) of ZHS; (**b**) band structure and (**d**) DOS of Cd@ZHS. The Fermi level is shifted to 0 eV.

According to computational optimization, the lattice constant increases from 7.7901 Å to 7.7972 Å with Cd doping. The distance between Cd and the neighboring oxygen atoms is significantly larger than that between Zn and O atoms in the undoped state. These results are consistent with the XRD data. Figure 11a reveals that ZHS is a semiconductor with a direct band gap of 3.852 eV, consistent with experimental measurements of approximately 4 eV [10,11,12]. As shown in Figure 11b, Cd@ZHS exhibits a semiconductor with an indirect band gap of 0.002 eV. Comparing the density of states (DOS) in Figure 11c,d reveals that Cd doping introduces DOS within the band gap of ZHS and reduces the band gap width. These findings suggest that Cd doping promotes electron transition from the valence band to the conduction band, significantly lowering the electrical resistivity of the ZHS sensor and improving its conductivity. These theoretical results are in good agreement with experimental observations.

**Figure 12:**
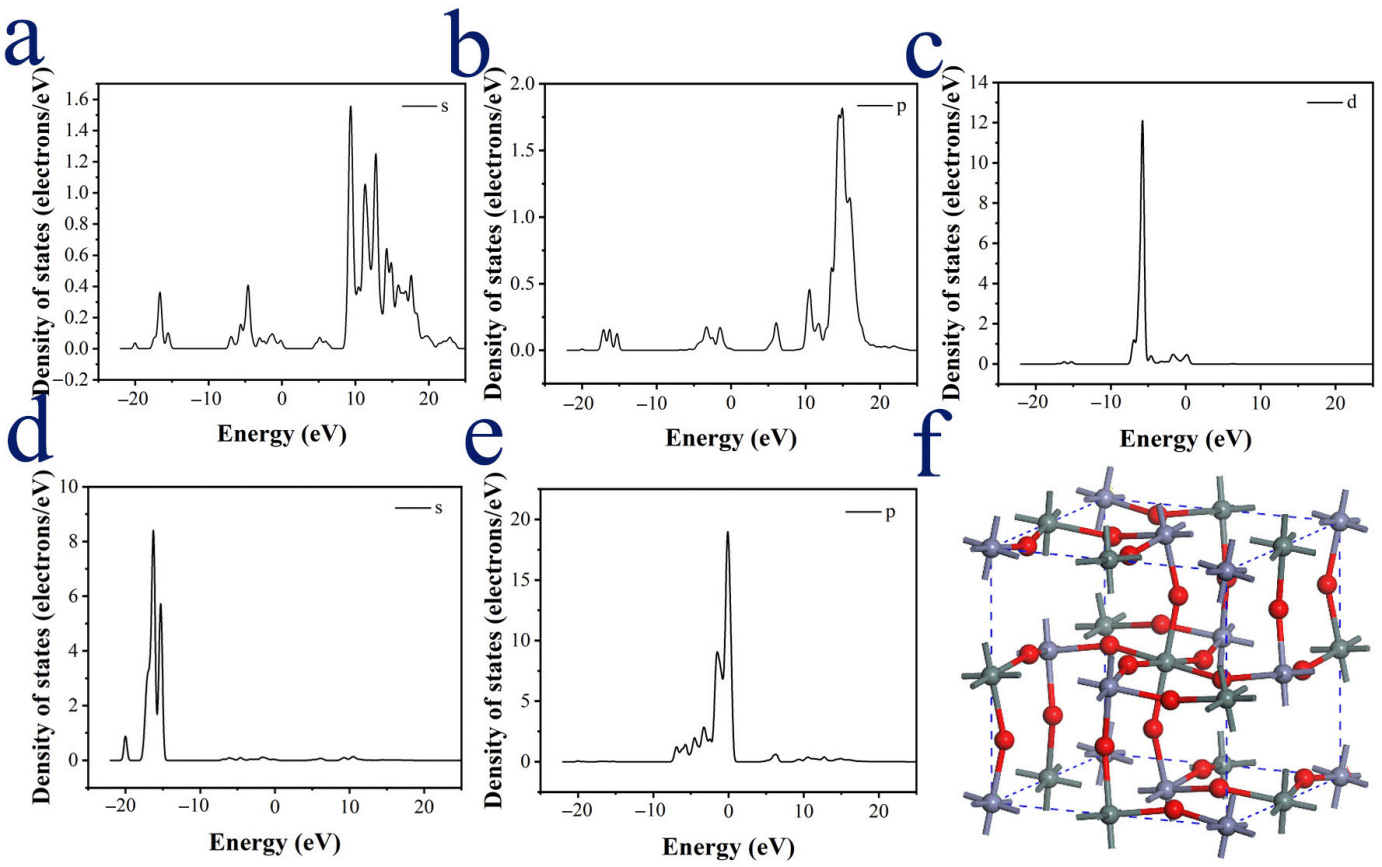
(**a**–**c**) Projected density of states (PDOS) of one Zn atom in lattice; (**d**,**e**) PDOS of six oxygen atoms connected with Zn; (**f**) ZHS lattice structure.

**Figure 13:**
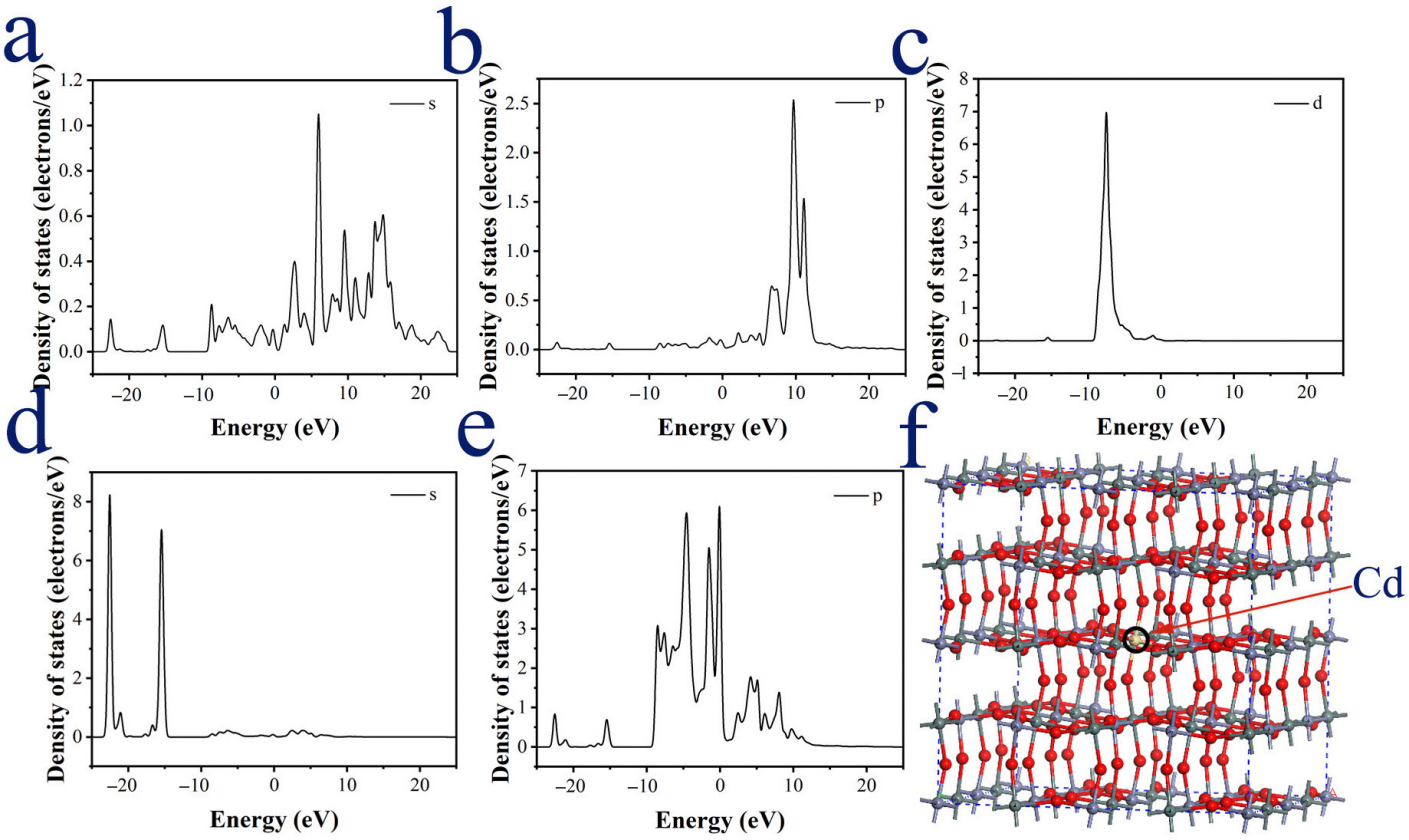
(**a**–**c**) Projected density of states (PDOS) of one Cd atom in 2×2×2 supercell lattice; (**d**,**e**) PDOS of six oxygen atoms connected with Cd; (**f**) Cd@ZHS 2×2×2 supercell lattice structure.

The projected density of states (PDOS) of Zn and its first neighboring O atoms in Figure 12 indicate that the bonds between Zn and its six neighboring O atoms are formed via the interactions of their s and p electrons in the valence bands. The PDOS of the Cd impurity and its first neighboring O atoms in Figure 13 reveal that the s and p orbital levels from the Cd impurity insert within the forbidden band of ZHS, forming a continuous energy level with the conduction band and thereby narrowing the band gap of ZHS. The bonding between the Cd impurity and its first neighboring O atoms is associated with electrons in s and p orbitals within the forbidden band and the conduction band, particularly the contributions of p orbital electrons in the conduction band.

### 3.2. Gas Sensing Enhancing Mechanism

**Figure 14:**
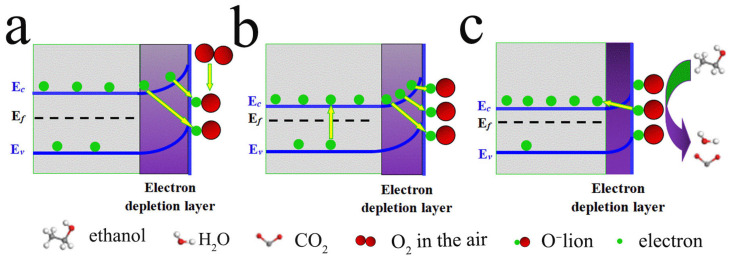
Schematic diagrams of gas-sensitive materials: (**a**) sketch of the energy band structure of ZHS in air; (**b**) energy band structure of Cd@ZHS; (**c**) Interaction of ethanol molecules with $O^{-}$ ions absorbed surface. $E_{c}$, $E_{f}$, and $E_{v}$ are the bottom of the conduction band, the Fermi level, and the top of the valance band, respectively.

The band structure of ZHS in air, as shown in Figure 14a, reveals that oxygen molecules adsorb electrons from the ZHS surface, thereby transforming into $O^{-}$ ions. The electron depletion on the ZHS surface elevates its potential energy, leading to an upward shift in the energy levels. As shown in previous studies [10,11,12] and corroborated by our theoretical calculations, ZHS exhibits a forbidden band width of approximately 4 eV. Figure 11 and Figure 13 illustrate that Cd doping introduces impurity energy levels within the forbidden band, which connect to the conduction band’s bottom, thereby narrowing the band gap. A reduced forbidden band width enhances the electron transition from the valence to conduction bands, increasing electron density in the conduction band and lowering resistance (as shown in Figure 10). Higher electron density produces more $O^{-}$ ions and reduces the depletion layer thickness on the ZHS surface. $O^{-}$ ions donate electrons back to the material when interacting with target gases. Consequently, a higher concentration of $O^{-}$ ions induces a greater resistance change during gas interaction, leading to an enhanced response value. After doping, the material’s resistance is significantly reduced, thereby reducing the sensor’s power consumption.

## 4. Conclusions

ZHS and Cd-doped ZHS samples were synthesized via a one-step hydrothermal method. After Cd doping, XRD peaks shifted to lower angles, and crystal particle sizes decreased, indicating increased lattice constants and altered crystallization behavior. Theoretical calculations revealed that Cd doping introduces impurity energy levels within the band gap, which connect to the lower portion of ZHS’s conduction band, thereby narrowing the band gap. A narrowed band gap enhances the probability of electron transitions from the valence band maximum to the conduction band minimum, increasing free electron density and $O^{-}$ ion adsorption on the ZHS surface. Consequently, the elevated $O^{-}$ ion concentration enhances the material’s responsiveness to ethanol gas.

## Data Availability

The original contributions presented in this study are included in the article. Further inquiries can be directed to the corresponding author.
